# First marine cryptotephra in Antarctica found in sediments of the western Ross Sea correlates with englacial tephras and climate records

**DOI:** 10.1038/s41598-019-47188-3

**Published:** 2019-07-23

**Authors:** Alessio Di Roberto, Ester Colizza, Paola Del Carlo, Maurizio Petrelli, Furio Finocchiaro, Gerhard Kuhn

**Affiliations:** 1 Istituto Nazionale di Geofisica e Vulcanologia, Sezione di Pisa, Via della Faggiola 32, 56126 Pisa, Italy; 2 Dipartimento di Matematica e Geoscienze, Università di Trieste, Via E. Weiss 2, 34127 Trieste, Italy; 3 Dipartimento di Fisica e Geologia, Università di Perugia, Via A. Pascoli, I-06123 Perugia, Italy; 4 Alfred-Wegener-Institut Helmholtz-Zentrum für Polar- und Meeresforschung, Am Alten Hafen 26, D-27568 Bremerhaven, Germany

**Keywords:** Volcanology, Palaeoclimate

## Abstract

We report the discovery of an important new cryptotephra within marine sediments close to Cape Hallett (northern Victoria Land), in the western Ross Sea, Antarctica. The cryptotephra is fully characterized for its texture, mineralogy and major- and trace-element data obtained on single glass shards. On the basis of geochemical composition, the cryptotephra is unequivocally correlated with the proximal deposits of an explosive eruption of the poorly known Mount Rittmann volcano, situated in northern Victoria Land. The cryptotephra is also correlated with a widespread tephra layer, which was erupted in 1254 C.E. and is present in numerous ice-cores and blue ice fields across East and West Antarctica. The characteristics of the tephra indicate that it was produced by a prolonged, moderate energy, mostly hydromagmatic eruption. This is the first time that a cryptotephra has been identified in marine sediments of the Ross Sea and in ice cores. It provides an important new and widespread stratigraphical datum with which the continental cryosphere and marine sedimentological records in Antarctica can be correlated. Moreover, from a purely volcanological point of view, the discovery further confirms the occurrence of a long-lasting, significant explosive eruption from Mount Rittmann in historical times that produced abundant widely dispersed fine ash. The study also highlights the inadequacy of current hazard assessments for poorly known volcanoes such as Mount Rittmann, located at high southern latitudes.

## Introduction

Volcanic ash produced during explosive eruptions may travel thousands of kilometers from the source and finally become deposited as layers invisible to the naked eye called cryptotephra. The discovery of cryptotephra is an important development in modern tephrochronology^1^; like macroscopic tephra, cryptotephra can be geochemically characterized and dated, and they represent invaluable isochrons for dating, correlating, and the synchronization of geological, paleoecological, paleoclimatic, and archeological records^1–8^. Compared with macroscopic tephra, a principal advantage of studying cryptotephra is that, because of their very fine grain size, they can be dispersed over much wider areas, even by relatively small-intensity eruptions, thus increasing the number of eruptions detectable and the number of tephra isochrons available for tephrochronological purposes^9^. Because of their greater visibility compared with the host ice, tephra and cryptotephra have been intensely exploited in Antarctic glacial archives^10–16^. Conversely, the study of tephra generally in marine sediment sequences of the Southern Ocean is still at a pioneering stage, and there are only a few dedicated studies^17–21^. Moreover, cryptotephra layers have never been identified in the Antarctic marine sediments.

## Study Area

**Figure 1:**
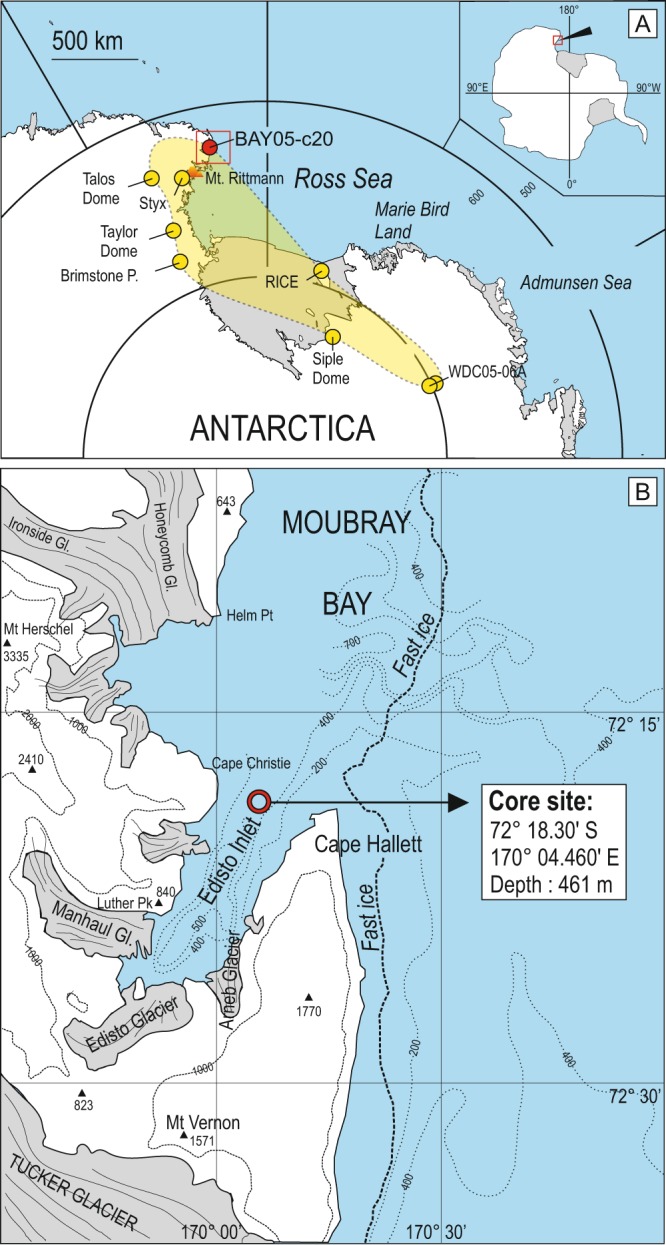
(**A**) Map of Antarctica showing the locations of BAY05-c20 core (red dot), Antarctic deep and shallow ice cores and blue ice field (yellow dots) where the 1254 C.E. tephra has been found. Yellow field correspond to the reconstructed dispersal area of 1254 C.E. tephra addressed in this study. (**B**) Location of core BAY05-c20. Bathymetric data and land morphology from map SS 58-60/2 (Cape Hallett), original scale 1:250,000 edited by U.S. Geological Survey^60^.

Edisto Inlet is small ice-filled fjord situated near Cape Hallett, in the western Ross Sea (Antarctica; Fig. 1A,B). It is approximately 15 km long and 4 km wide, elongated NNE-SSW, with a maximum water depth of approximately 500 m and a sill 400 m deep divides the fjord from Moubray Bay to the north. Several small glaciers flow into the inlet: Manhaul Glacier in the west, and Edisto and Arneb glaciers in the south and east, respectively (Fig. 1B). Sediment cores and geophysical data acquired near the fjord entrance during the 2002 and 2005 PNRA (National Antarctic Research Program) Italian expeditions highlighted a generally flat seabed morphology with an elongated bedrock ridge in the axis zone (Fig. 1B). Sediment cores from the basin recovered a very expanded Holocene sequence characterized by soft biogenic laminated sediments. The lamination corresponds to several bloom cycles of diatoms species^22,23^. The thick Holocene sequence contains a very detailed Holocene paleoenvironmental and paleoclimatic record for the last 4.5 ka that is undergoing investigation.

## Results

**Figure 2:**
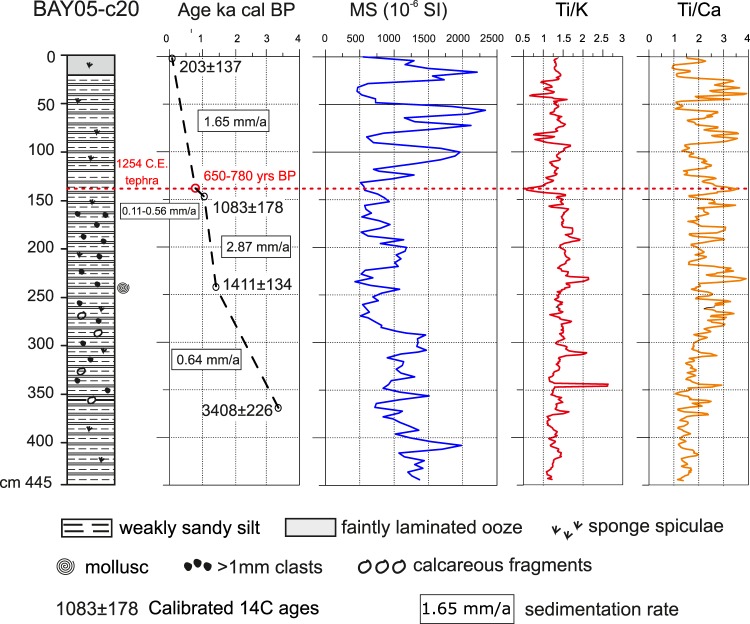
Lithology, age model, magnetic susceptibility curve and XRF representative data (element ratios) for core BAY05-c20. Red dashed line highlights the position of BAY05-c20/139-140 cryptotephra. Partially redraw after^23^.

A cryptotephra was identified at 139–140 cm depth in core BAY05-20c (Fig. 2) using procedures described below (see Methods section). The cryptotephra in core BAY05-c20 do not show obvious, extensive disturbance by reworking. It is entirely made of volcanic particles like glass shards and micropumices, with minor non-volcanic (mostly biogenic) components. Volcanic particles show pristine morphologies with preserved fragile glass tips, have a well‐sorted grainsize distribution (Fig. 2), and are characterized by very homogeneous geochemical and mineralogical composition. Very sharp geochemical signal in XRF data (ex. Ti/K and Ti/Ca ratios in Fig. 2) indicate that shards are vertical distributed in a very narrow sediment interval. These are all good indicators of the primary nature of the cryptotephra layer^24–28^.

**Figure 3:**
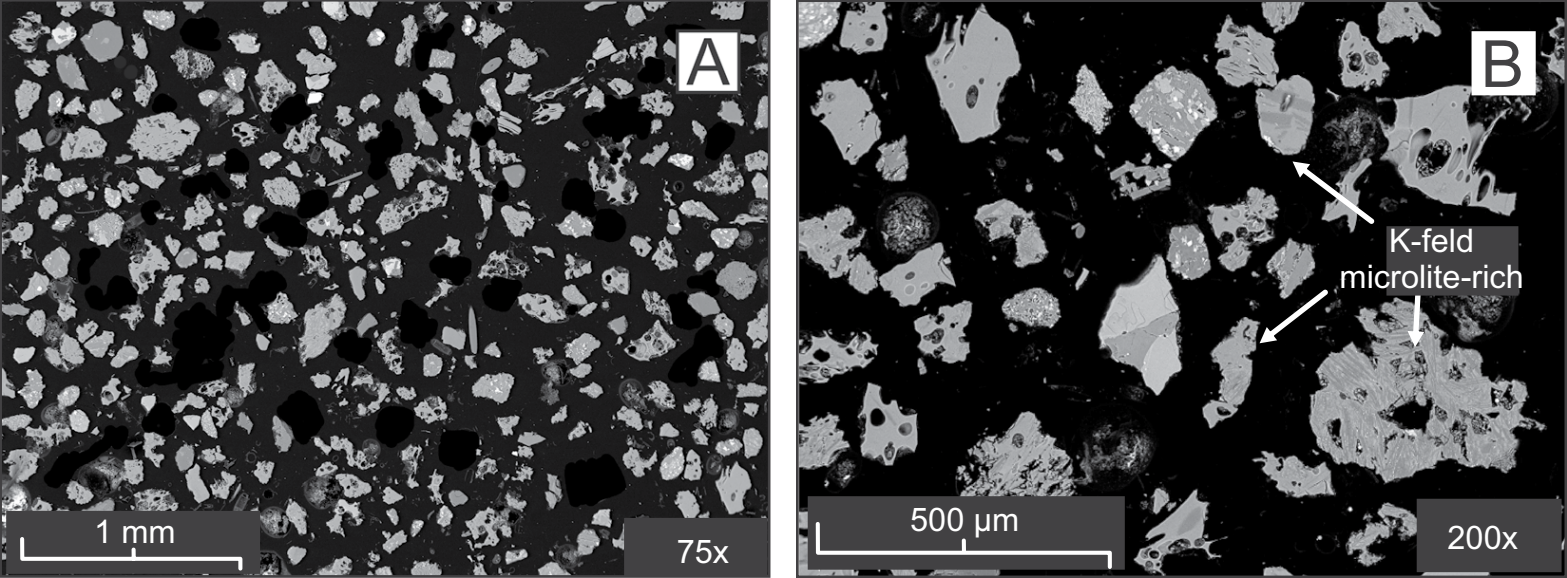
SEM backscatter images of glass particles forming the BAY05-c20/139-140 cryptotephra.

**Figure 4:**
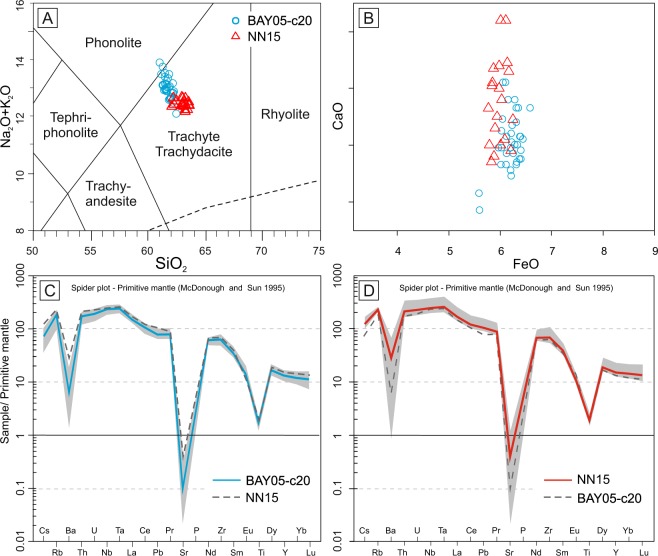
(**A**) Total alkali-silica (TAS) and (**B**) CaO/FeO diagrams showing the glass composition of BAY05-c20/139-140 cryptotephra and of NN15 trachytic pumice from Mount Rittmann. (**C**,**D**) Primitive mantle (PM) normalized spider diagram^31^ showing the trace element distribution of BAY05-c20/139-140 cryptotephra and of NN15 sample.

The BAY05-c20/139-140 cryptotephra consists of well-sorted, colorless to light-green glass shards up to 200 µm and micropumices up to 500 µm, respectively; scarce light-brown glass fragments are also present (Fig. 3). The micropumices are moderately to highly vesicular and range texturally from frothy-vesicular to tubular, while the shards are dense to poorly vesicular and blocky, y-shaped and bubble wall-shaped (Fig. 3A,B). Approximately half of the particles are aphyric and the remainder are rich in acicular microliths of k-feldspar, plagioclase and Fe-Ti spinels (Fig. 3B). Crystals of feldspars and clinopyroxene also occur in the cryptotephra and are rimmed by volcanic glass, together with minor, slightly altered to palagonitized sideromelane and holocrystalline volcanic rocks. Glass compositions of the BAY05-c20/139-140 tephra are predominantly trachytic but plot across the boundary with phonolites in the total alkalis-silica diagram (TAS^29^ Fig. 4A). Total alkalis values range from 11.9 to 13.6 wt% and K_2_O/Na_2_O ratios average 0.62 (σ1 = 0.03). Mean CaO and FeO contents are ~1 wt% (σ1 = 0.1) and ~6.1 wt% (σ1 = 0.2), respectively (Fig. 4B). To further determine the origin of the BAY05-c20/139-140 tephra we also analysed the glass composition in trachyte pumices sampled from the rim of the Mount Rittmann caldera (Supplemental Fig. 1). The latter (sample NN15^30^) are highly vesicular, aphyric pumice fragments representing the matrix of a lag breccia containing abundant basement rocks. The glass composition of NN15 is uniformly trachytic, overlapping with the alkalis-poor end of the range of values for BAY05-c20/139-140 (Fig. 4A). NN15 has an average value for total alkalis of 12.46 wt% (σ1 = 0.28), and K_2_O/Na_2_O = 0.68 (σ1 = 0.03). CaO and FeO average values are 1.15 wt% (σ1 = 0.1) and 6 wt% (σ1 = 0.1), respectively, and are remarkably similar to BAY05-c20/139-140 (Fig. 4B). Trace element distributions for BAY05-c20/139-140 and NN15 pumices are reported as primitive mantle (PM) normalized diagrams^31^ in Fig. 4C and D, respectively. The spidergram patterns are remarkably alike, with concentrations that are enriched compared to PM values for most of the investigated elements (i.e., from ~10x to ~300x^31^). Significant negative anomalies are evident for Ba, Sr, and Ti, with mean PM normalized concentrations of ~5, ~0.1, and ~2, respectively.

Average major oxide and trace elements compositions for BAY05-c20/139-140 and NN15 are presented in Supplementary Table 1. Complete analyses and reference standards are given in Supplementary Table 2.

## Discussion

### Cryptotephra emplacement

Determining whether a tephra layer is primary or has undergone secondary transport processes or is the result of erosion and reworking of old volcanic deposits is of crucial to assess its value as potential tephrochronological and stratigraphic marker useful for the synchronization and correlation of geological archives. Many studies have addressed the study of tephra and cryptotephra in the marine sediment sequences of the north hemisphere with relevant discussion on their preservation and on issues pertaining to taphonomy and interpretation^24,28,32–37^.

**Figure 5:**
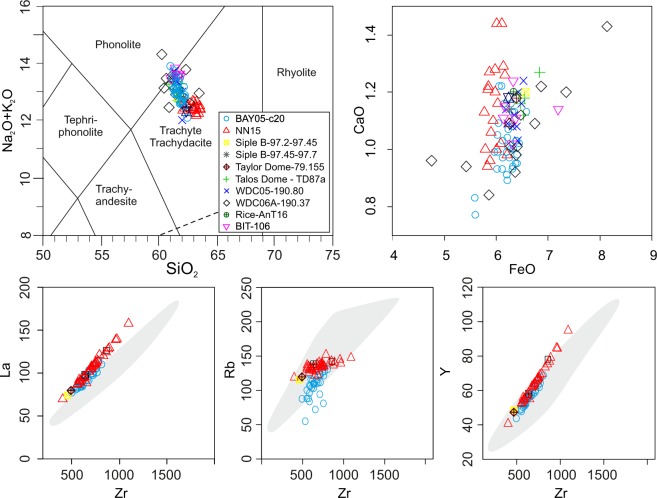
Comparison between glass composition of BAY05-c20/139-140 cryptotephra and NN15 sample with and those of 1254 C.E. tephra layers sampled at different location of East and West Antarctica. In gray the bulk rock compositional field of Mount Rittmann^30^.

As mentioned above BAY05-c20 sediment cores collected a very expanded Holocene sequence almost entirely made by soft biogenic laminated sediments^22,23^ without evidence for consistent ice-rafted debris (IRD) input in the sediment interval where the cryptotephra is contained. Thus, the sedimentation via iceberg rafting^28,32,35^ for the studied cryptotephra can reasonably be ruled out. Based on the well-sorted grainsize distribution of ash (Fig. 2A,B), and their strong geochemical homogeneity (Figs 4 and 5) two mechanisms can thus be invoked for the deposition of BAY05-c20/139-140 cryptotephra i.e. the fall-out from an eruptive column and sea-ice rafting. These mechanisms involve the rapid transfer of the volcanic ash from the eruption plume towards the marine sediments. In the second case, considering that the area of Cape Hallett is characterized exclusively by sea ice, the interval between the emission of the ash and its final sedimentation can be in the order of a few months which is the duration of seasonal sea ice cycle. Therefore, in both cases, the significance of BAY05-c20/139-140 cryptotephra as isochronous horizons for the synchronization and correlation of ice-core records and marine sequences is maintained.

### Source correlation

The main sources of Antarctic tephra are the South Sandwich Islands, followed by intraplate Antarctic volcanoes in Marie Byrd Land and Victoria Land. Extra-Antarctic sources, mainly South America volcanoes, have been also invoked^13^ but most remain to be confirmed^38^. Presently, the only confirmed extra-Antarctic tephra in ice-core records are a cryptotephra attributed to the Oruanui super-eruption from Taupo volcano, dated at ca. 25.6 ka^39^, and extremely fine ash attributed to a Puyehue‐Cordón Caulle (Chile) eruption in 2011^40^ and to a Samalas (Indonesia) eruption in 1259 C.E. Based on the relatively coarse grain size (indicating a relatively proximal source) and the evolved alkaline phonolitic—trachytic composition of BAY05-c20/139-140, a correlation is excluded with the low-alkali tholeiitic South Sandwich Islands, calc-alkaline South Shetland volcanoes and extra-Antarctic (South Americana and New Zealand) volcanoes as potential sources. Conversely, intraplate alkaline volcanoes of the West Antarctic rift system are compositionally better candidates and geographically closer, comprising volcanoes of the McMurdo Volcanic Group in Victoria Land and those in Marie Byrd Land^41^. The closest compositional similarity is with a widespread trachytic tephra conventionally referred as the 1254 C.E. tephra (Figs 4 and 5, and Supplementary Table 1), found in deep ice cores at Taylor Dome^42^, Siple Dome and Talos Dome^12,13,43^, and several shallow ice cores in East and West Antarctica (Styx glacier, RICE, WDC05 and WDC06A^44^). A good match, also exists with BIT106, an englacial tephra layer sampled in blue ice at Brimstone Peak (northern Victoria Land - NVL), which is also correlated with the 1254 C.E. tephra (Supplementary Table 1)^42^. Besides being very similar from a geochemical point of view, the BAY05-c20/139-140 cryptotephra has components, mineralogical and textural characteristics identical to those of the 1254 C.E. tephra found in ice-cores records (Supplement Table 3), which further support their correlation. Only recently the Mount Rittmann volcano was proposed as the most probable source for all the above mentioned tephra deriving from the 1254 C.E. eruption^13,45^; this is confirmed here by the strong statistical correlation between the glass composition of BAY05-c20/139-140 and that of NN15 from summit part of Mount Rittmann (Figs 4 and 5, and Supplementary Table 1). Results of a statistical comparison between BAY05-c20/139-140 tephra, NN15 pumices from Mount Rittmann and 1254 C.E. tephra samples are provided in Supplementary Table 1. They demonstrate that all the analyzed samples have remarkably similar chemical compositions and thus can be considered as correlatives. However, the strong chemical similarity between the proximal tephra sample NN15 and the 1254 C.E. tephra (including the BAY05-c20/139-140 cryptotephra), does not necessarily imply that they have been emplaced by the same eruption, but instead that they derive from the same volcanic source.

### Age

It was not possible to directly date the BAY05-c20/139-140 cryptotephra using radiometric methods due to the fine grain size and low abundance of datable material (e.g. feldspar crystals). However, the age of 1254 C.E. tephra (and consequently of the BAY05-c20/139-140 cryptotephra), has been determined by glacial proxies in Talos Dome (TD87a) at 696 ± 2 yrs BP^13^, in Taylor Dome (79.155) at 709 ± 71 yrs BP^46^, in Siple Dome B (97.7) at 675 ± 25 yrs before 1996^46^, and in WDC05 and WDC06A ice cores (at 190.8 and 190.37 m, respectively) at 687 ± 7 yrs cal BP^43,44^. By considering age determination uncertainties we argue that the time interval during which the 1254 C.E. tephra emplaced, ranges between 650 and 780 yrs BP.

The age model available for core BAY05-c20 (WRS-CH in^23^) was published by^23^ and is based on three accelerator mass spectrometry (AMS) radiocarbon ages measured on acid insoluble organic matter from bulk sediment samples, and one mollusc shell. The down-core dates were corrected by subtracting the box-core top age, which embed the regional marine reservoir effect and the local dead carbon contamination, assuming these did not change over the Holocene^23^. The corrected dates were then converted into calibrated ages using the CALIB REV 7.1.0 program and the MARINE13 calibration curve. The ages between dated levels were interpolated, assuming a linear sedimentation rate^23^.

The BAY05-c20 marine core chronologys can be updated by introducing and integrating the age determined for 1254 C.E. tephra which in this sequence is at 139–140 cm of sediment depth (Fig. 2). Consequently, the sedimentation rate for the top ~150 cm of the sequence is not linear as previously suggested by^23^ and at least for the 139–147 cm interval it varies between 0.11–0.56 mm/yrs.

### Clues to eruption dynamics and intensity

The characteristics of tephra, including grain size, particle morphology and texture, and tephra dispersal provide information on magma fragmentation mechanisms, eruption dynamics and eruption intensity. According to the descriptions available in the literature, the 1254 C.E. tephra always occurs in a low concentration with a diffuse appearance and it contains variable amounts of <200 µm volcanic shards^42–44,47,48^. Discrete, coarser-grained layers have never been found, even in outcrops closer to Mount Rittmann. The 1254 C.E. tephra has an extremely wide dispersal; in fact, if we include the new site of the Edisto Inlet with those previously known^44^, we obtain a dispersal area of >950 000 km^2^ (Fig. 1A), with the tephra occurrences dispersed at 360° around Mount Rittmann and reaching a distance >2000 km from the source. This is only a minimum estimate and it may be further enlarged by studying ice-cores drilled west of the Taylor Dome site (which is the westernmost site where the tephra has been found), or by studying cryptotephra in sediment cores from the Ross Sea, east of Mount Rittmann.

Features of the 1254 C.E. tephra, such as particle fine grainsize and shape, indicate that the associated eruption was characterized by a very efficient magma fragmentation. However, the eruption intensity was not enough to produce a discrete layer even in outcrops situated proximal to the source volcano. The eruption was possibly characterized by a sustained and prolonged emission of ash. The fine grain size facilitated a wide dispersal pattern through 360° by winds, which repeatedly changed direction during the eruption. Northwesterly winds transported most of the ash towards the southeast, resulting in dispersal over NVL to the WAIS (West Antarctic Ice Divide). Had the eruption been a higher intensity Plinian eruption, the mean grain size and deposit thickness would be expected to be higher, at least in proximal to medial sites.

This hypothesis is somewhat supported by trace element geochemical data presented by^44^. That author pointed out that compositional variabilities exist between samples of 1254 C.E. tephra recovered from coastal areas and those sampled more inland. According to^44^ these differences suggest that the eruption was fed by a zoned magma and that winds could have differentially transported products from the early phases of the eruption in areas close to the coast of Antarctica, with products from a later phase of the eruption deposited more inland. This wouldn’t be possible for a short-lived eruption fed by a relatively small and homogeneous magma batch. An example of eruptive activity with similar characteristics is the 14 April - 22 May 2010 summit eruption of Eyjafjallajökull, Iceland. This relatively small sub-Plinian event (VEI = 3) was characterized by a sustained character and quite long duration (weeks), and was able to disperse fine ash over thousands of kilometers including large parts of the Europe^49^. However, it produced a deposit of coarse ash only very close (2 km) to the vent^50^.

### Conclusions and perspectives

Having considered the results of our analyses we conclude that BAY05-c20/139-140 cryptotephra is a primary tephra horizon correlated with the 1254 C.E. tephra found in ice-cores and blue-ice records over the NVL to the WAIS divide. Thus, it can be utilized as marker bed for the correlation and synchronization of Antarctic records and as isochronous horizons for the linking of paleoclimatic/paleoenvironmental sequences.

The first identification of a cryptotephra in Holocene marine sediments of Cape Hallett is an important event in the study of tephra in Antarctica and opens up Antarctica to the cryptotephra revolution^1^. It demonstrates that cryptotephra layers can be preserved not only in continental ice records but also in marine sediment sequences in Antarctica (and elsewhere). Future studies of marine sediment sequences focused on the identification and characterization of cryptotephra may expand the potential and application of tephrochronology in the region by increasing the number of eruptions detected, of any intensity, and using them as tephrostratigraphic markers. The discovery of the 1254 C.E. cryptotephra in sediments at Edisto Inlet now permits, for the first time in Antarctica, the unequivocal, independent time-stratigraphic correlation and synchronization between continental ice-archives and marine sediments, which is fundamental for understanding the nature of connections and coupling processes between atmospheric, ice-sheets, ocean dynamics, marine sedimentary systems and climate change.

From a purely volcanological perspective, this discovery also confirms and reinforces the idea that volcanoes of NVL, and in particular Mount Rittmann, have had a high-level of eruptive activity during the Holocene, with the Mount Rittmann eruption probably representing at least one long-lasting eruption in historical time. During this eruption large amounts of fine volcanic ash were ejected and spread over large part of East and West Antarctica, from Cape Hallett to WAIS (>950,000 km^2^). It may prove to be one of the most important time-stratigraphic markers for Antarctica.

The scientific community has only recently paid some attention to the potential volcanic hazards posed by, and the greater-than-expected impact of, ash-forming eruptions occurring from volcanoes located at high southern latitudes^51^. Those researchers concluded that the potential impact connected with volcanic ash from Antarctic eruptions is mainly linked to a combination of volcano location and eruption column height. Models show that eruptions with higher plumes (>10 km) from volcanoes located at latitudes <70° have a higher potential to spread ash out of Antarctica^51^. An eruption like that responsible for the 1254 C.E. tephra would easily reach a plume height >10 km and keep it aloft for days to weeks. It is thus clear that such eruptions from high southern latitude volcanoes need proper hazard assessments, including a much better understanding and numerical modelling of ash dispersal. Better defining the volcanic risk of Mount Rittmann (and also for other volcanoes, such as Mount Melbourne) would also benefit the numerous Antarctic research bases (e.g. Jang Bogo station (Korea), Gondwana station (Germany) and Mario Zucchelli station (Italy)) that are within easy reach of significant ash fallout, and which would be affected during a future eruption.

## Materials and Methods

The core BAY05-20c was collected during the XX° PNRA Expedition (2004–2005), in the framework of 2004/4.10 a – Bay Project situated in the Western Ross Sea, on the continental shelf at Cape Hallett (Fig. 1A). The marine diatom records and the age models are based on calibrated ^14^C dates performed on acid insoluble organic matter, and one mollusc of the BAY05-20c cores has recently been published by^23^. The cryptotephra layer was identified by observing with a microscope sediments that displayed significant peaks in magnetic susceptibility curve, together with associated indicative features shown by X-ray fluorescence (XRF) core scanning data (Fig. 2). In addition, the core BAY05-c20 was sampled every 4 cm with a mean temporal resolution of ~20 years from 1 to 240 cm (0.1–1.4 ka) for diatom biostratigraphy^23^ and no glass concentration peak have been identified beside at 139–140 cm, in correspondence of the cryptotephra. Bulk tephra was impregnated in epoxy resin to perform textural, mineralogical and geochemical characterizations. The textures of glass particles and mineral phase composition were studied at the Istituto Nazionale di Geofisica e Vulcanologia, Sezione di Pisa (INGV-Pisa) using a scanning electron microscope (SEM), Zeiss EVO MA. Major and minor element glass composition was determined using a JEOL JXA-8200 electron microprobe (EPMA) equipped with five wavelength-dispersive spectrometers at the High-Pressure High-Temperature (HPHT) Laboratory of INGV-Rome. Operating conditions were 15 kV accelerating voltage, 8 nA beam current, 5 mm probe diameter, 10 and 5s acquisition time for peak and background, respectively). Standards of glass were analysed to test the accuracy of data during the EPMA analyses (see Supplementary Table 2). The trace element compositions of glass shards were determined using laser ablation inductively coupled plasma mass spectrometry (LA-ICP-MS) system at the Department of Physics and Geology, University of Perugia (Italy). The analyses were performed with a Teledyne Photon Machine G2 laser ablation system coupled with a Thermo Fisher Scientific iCAP-Q quadrupole-based ICP-MS^52,53^. The operating conditions were optimized by analysing reference material^54^ NIST SRM 612 to provide maximum signal intensity and stability for the ions of interest while suppressing oxides formation (ThO^+/^Th^+^ below 0.5%). The U/Th ratio was also monitored and maintained close to 1. The stability of the system was evaluated on ^139^La, ^208^Pb, ^232^Th, and ^238^U by a short-term stability test. It consisted of 5 acquisitions (one minute each) on a linear scan of NIST SRM 612 glass reference material^52,53^. Tephra glasses were analysed by using a circular laser beam with a diameter of 15 µm (BAY05-c20-139-140), and 25 µm (NN15), a frequency of 8 Hz, and an energy density at the sample surface of 3.5 J/cm^2^. NIST SRM 610 reference material^54^ was used as the calibrator and ^29^Si as the internal standard. USGS BCR2G reference material was analysed as unknown to provide a quality control^55^ (Supplemental Table 2). Under these operating conditions, precision and accuracy are better than 10% for all the investigated elements^52,53^. The geochemical composition of the cryptotephra was compared with a database of major- and trace-element compositions of Neogene-Quaternary tephra from Antarctic ice cores, marine sediments, blue ice and continental outcrops as well as tephra cropping out in volcanic sources in Antarctica and circum-Antarctic areas^38^. Similarity Coefficient index (SC)^56,57^ and statistical distance (D^2^)^58,59^ were used to assess the potential correlations. The SC is calculated as the average of ratios of elemental concentrations between pairs of compositional analyses of tephra (calculated as averages). Two samples are considered as correlatives if they have a SC value close to 1, with SC > 0.92 considered as a lower bounding value for a good statistical correlation. By contrast, statistical distance (D^2^) measures the difference between two tephra based on their average compositions and standard deviations^58,59^. D^2^ is a distance function, thus the lower its value the higher is the similarity between two samples being compared^59^. Compositionally identical samples have D^2^ = 0. The use of these SC and D^2^ indices is not without problems but in general the indices are widely used and give a broad numerical-strength indication of the compositional similarity between two samples.

## Supplementary information

Supplemental figure 1

Supplemental Table 1

Supplemental Table 2

Supplemental Table 3

## Data Availability

All data generated or analysed during this study are included in this published article (and its Supplementary Information files).

## References

[1] Davies SM (2015). Cryptotephras: The revolution in correlation and precision dating. J. Quat. Sci..

[2] Lowe DJ (2011). Tephrochronology and its application: A review. Quaternary Geochronology.

[3] Riede F, Thastrup MB (2013). Tephra, tephrochronology and archaeology - a (re-)view from Northern. Europe. Herit. Sci..

[4] Lane CS, Cullen VL, White D, Bramham-Law CWF, Smith VC (2014). Cryptotephra as a dating and correlation tool in archaeology. Journal of Archaeological Science.

[5] Lane CS, Lowe DJ, Blockley SPE, Suzuki T, Smith VC (2017). Advancing tephrochronology as a global dating tool: Applications in volcanology, archaeology, and palaeoclimatic research. Quaternary Geochronology.

[6] Ponomareva V, Portnyagin M, Davies SM (2015). Tephra without Borders: Far-Reaching Clues into Past Explosive Eruptions. Front. Earth Sci..

[7] Lowe DJ (2017). Correlating tephras and cryptotephras using glass compositional analyses and numerical and statistical methods: Review and evaluation. Quaternary Science Reviews.

[8] Di Roberto A (2018). Tephra and cryptotephra in a~60,000-year-old lacustrine sequence from the fucino basin: New insights into the major explosive events in Italy. Bull. Volcanol..

[9] Lowe John J., Ramsey Christopher Bronk, Housley Rupert A., Lane Christine S., Tomlinson Emma L. (2015). The RESET project: constructing a European tephra lattice for refined synchronisation of environmental and archaeological events during the last c. 100 ka. Quaternary Science Reviews.

[10] Colizza E, Finocchiaro F, Marinoni L, Menegazzo Vitturi L, Brambati A (2003). Tephra Evidence in Marine Sediments from the Shelf of the Western Ross Sea. Terra Antarct..

[11] Palais JM, Kyle PR, Mosley‐Thompson E, Thomas E (1987). Correlation of a 3,200 year old tephra in ice cores from Vostok and South Pole Stations, Antarctica. Geophys. Res. Lett..

[12] Dunbar NW, Kurbatov AV (2011). Tephrochronology of the Siple Dome ice core, West Antarctica: Correlations and sources. Quat. Sci. Rev..

[13] Narcisi B, Petit JR, Delmonte B, Scarchilli C, Stenni B (2012). A 16,000-yr tephra framework for the Antarctic ice sheet: A contribution from the new Talos Dome core. Quat. Sci. Rev..

[14] Narcisi B, Petit JR, Langone A (2017). Last glacial tephra layers in the Talos Dome ice core (peripheral East Antarctic Plateau), with implications for chronostratigraphic correlations and regional volcanic history. Quat. Sci. Rev..

[15] Riggs NR, Duffield WA (2008). Record of complex scoria cone eruptive activity at Red Mountain, Arizona, USA, and implications for monogenetic mafic volcanoes. J. Volcanol. Geotherm. Res..

[16] McConnell JR (2017). Synchronous volcanic eruptions and abrupt climate change ∼17.7 ka plausibly linked by stratospheric ozone depletion. Proc. Natl. Acad. Sci..

[17] Moreton SG, Smellie JL (1998). Identification and correlation of distal tephra layers in deep-sea sediment cores, Scotia Sea, Antarctica. Ann. Glaciol..

[18] Fretzdorff S, Smellie JL (2002). Electron microprobe characterization of ash layers in sediments from the central Bransfield basin (Antarctic Peninsula): Evidence for at least two volcanic sources. Antarct. Sci..

[19] Aymerich IF, Oliva M, Giralt S, Martin-Herrero J (2016). Detection of tephra layers in antarctic sediment cores with hyperspectral imaging. PLoS One.

[20] Oppedal LT, van der Bilt WGM, Balascio NL, Bakke J (2018). Patagonian ash on sub-Antarctic South Georgia: expanding the tephrostratigraphy of southern South America into the Atlantic sector of the Southern Ocean. J. Quat. Sci..

[21] Hillenbrand, C., Moreton, S. G., Caburlotto, A., Pudsey, C. J. & Lucchi, R. G. Volcanic time-markers for Marine Isotopic Stages 6 and 5 in Southern Ocean sediments and Antarctic ice cores: implications for tephra correlations between palaeoclimatic records. **27**, 518–540 (2008).

[22] Finocchiaro F (2005). Record of the early Holocene warming in a laminated sediment core from Cape Hallett Bay (Northern Victoria Land, Antarctica). in. Global and Planetary Change.

[23] Mezgec K (2017). Holocene sea ice variability driven by wind and polynya efficiency in the Ross Sea. Nat. Commun..

[24] Bourne AJ (2010). Distal tephra record for the last ca 105,000 years from core PRAD 1-2 in the central Adriatic Sea: Implications for marine tephrostratigraphy. Quat. Sci. Rev..

[25] Gudmundsdóttir ER, Eiríksson J, Larsen G (2011). Identification and definition of primary and reworked tephra in Late Glacial and Holocene marine shelf sediments off North Iceland. J. Quat. Sci..

[26] Gudmundsdóttir ER, Larsen G, Eiríksson J (2011). Two new Icelandic tephra markers: The Hekla Ö tephra layer, 6060 cal. yr BP, and Hekla DH tephra layer, ~6650 cal. yr BP. land-sea correlation of mid-Holocene tephra markers. Holocene.

[27] Griggs AJ, Davies SM, Abbott PM, Rasmussen TL, Palmer AP (2014). Optimising the use of marine tephrochronology in the North Atlantic: A detailed investigation of the Faroe Marine Ash Zones II, III and IV. Quat. Sci. Rev..

[28] Abbott PM, Austin WEN, Davies SM, Pearce NJG, Hibbert FD (2013). Cryptotephrochronology of the Eemian and the last interglacial-glacial transition in the North East Atlantic. J. Quat. Sci..

[29] Le Bas MJL, Maitre RWL, Streckeisen A, Zanettin B (1986). A chemical classification of volcanic rocks based on the total alkali-silica diagram. J. Petrol..

[30] Armienti P, Tripodo A (1991). Petrography and chemistry of lavas and comagmatic xenoliths of Mt. Rittmann, a volcano discovered during the IV Italian expedition in Northern Victoria Land (Antarctica). Mem. della Soc. Geol. Ital..

[31] McDonough WF, Sun Ss (1995). The composition of the Earth. Chem. Geol..

[32] Brendryen J, Haflidason H, Sejrup HP (2010). Norwegian Sea tephrostratigraphy of marine isotope stages 4 and 5: Prospects and problems for tephrochronology in the North Atlantic region. Quat. Sci. Rev..

[33] Abbott PM, Davies SM, Austin WEN, Pearce NJG, Hibbert FD (2011). Identification of cryptotephra horizons in a North East Atlantic marine record spanning marine isotope stages 4 and 5a (∼60,000-82,000 a b2k). Quat. Int..

[34] Cage AG, Davies SM, Wastegård S, Austin WEN (2011). Identification of the Icelandic Landnám tephra (AD 871 ± 2) in Scottish fjordic sediment. Quat. Int..

[35] Austin WEN, Abbott PM, Davies S, Pearce NJG, Wastegård S (2014). Marine tephrochronology: an introduction to tracing time in the ocean. Geol. Soc. London, Spec. Publ..

[36] Lowe TDJ, Alloway B, Sciences O (2014). Zealand, N. & Sciences, E. *Encyclopedia of Scientific Dating*. Methods. Encyclopedia of Scientific Dating Methods.

[37] Jennings A (2014). Holocene tephra from Iceland and Alaska in SE Greenland Shelf Sediments. Geol. Soc. London, Spec. Publ..

[38] Del Carlo P (2018). Late Glacial-Holocene tephra from southern Patagonia and Tierra del Fuego (Argentina, Chile): A complete textural and geochemical fingerprinting for distal correlations in the Southern Hemisphere. Quat. Sci. Rev..

[39] Dunbar NW (2017). New Zealand supereruption provides time marker for the Last Glacial Maximum in Antarctica. Sci. Rep..

[40] Koffman BG (2017). Rapid transport of ash and sulfate from the 2011 Puyehue-Cordón Caulle (Chile) eruption to West Antarctica. J. Geophys. Res. Atmos..

[41] LeMasurier W.E., Thomson J.W., Baker P.E., Kyle P.R., Rowley P.D., Smellie J.L., Verwoerd W.J. (1990). Volcanoes of the Antarctic Plate and Southern Oceans.

[42] Dunbar NW (2003). Tephra layers in the Siple Dome and Taylor Dome ice cores. J. Geophys. Res..

[43] Narcisi B, Petit JR, Chappellaz J (2010). A 70 ka record of explosive eruptions from the TALDICE ice core (Talos Dome, East Antarctic plateau). J. Quat. Sci..

[44] Iverson, N. A. Characterization and correlation of englacial tephra from blue ice areas and ice cores, Antarctica Nels Anton Iverson Dissertation Submitted as Partial Fulfillment of the Requirements for the Degree of Doctor of Philosophy in Earth and Environmental Sc (2017).

[45] Kyle, P. R. & Lee, M. J. Crossing New Frontiers - Tephra Hunt in Transylvania 24-29 June 2018 in Romania, Europe. 16185 (2018).

[46] Hawley RL, Waddington ED, Alley RB, Taylor KC (2003). Annual layers in polar firn detected by Borehole Optical Stratigraphy. Geophys. Res. Lett..

[47] Dunbar, N. *Blue Ice Tephra II - Brimstone Peak*, 10.7265/N5MG7MDK (2003).

[48] Iverson NA, Kalteyer D, Dunbar NW, Kurbatov A, Yates M (2017). Quaternary Geochronology Advancements and best practices for analysis and correlation of tephra and cryptotephra in ice. Quat. Geochronol..

[49] Gudmundsson MT (2012). Ash generation and distribution from the April-May 2010 eruption of Eyjafjallajökull, Iceland. Sci. Rep..

[50] Bonadonna C (2011). Tephra sedimentation during the 2010 Eyjafjallajkull eruption (Iceland) from deposit, radar, and satellite observations. J. Geophys. Res. Solid Earth.

[51] Geyer A, Marti A, Giralt S, Folch A (2017). Potential ash impact from Antarctic volcanoes: Insights from Deception Island’s most recent eruption. Sci. Rep..

[52] Petrelli M, Laeger K, Perugini D (2016). High spatial resolution trace element determination of geological samples by laser ablation quadrupole plasma mass spectrometry: implications for glass analysis in volcanic products. Geosci. J..

[53] Petrelli M, Morgavi D, Vetere F, Perugini D (2016). Elemental imaging and petro-volcanological applications of an improved Laser Ablation Inductively Coupled Quadrupole Plasma Mass Spectrometry. Periodico di Mineralogia.

[54] Pearce NJG (1997). A compilation of new and published major and trace element data for NIST SRM 610 and NIST SRM 612 glass reference materials. Geostand. Newsl..

[55] Jochum KP (2005). MPI-DING glasses: New geological reference materials for *in situ* Pb isotope analysis. Geochemistry, Geophys. Geosystems.

[56] Borchardt GA, Harward ME, Schmitt RA (1971). Correlation of volcanic ash deposits by activation analysis of glass separates. Quat. Res..

[57] Sarna-Wojcicki, A. M., Morrison, S. D., Meyer, C. E. & Hillhouse, J. W. Correlation of upper Cenozoic tephra layers between sediments of the western United States and eastern Pacific Ocean and comparison with biostratigraphic and magnetostratigraphic age data. *Geol. Soc. Am. Bull*, 10.1130/0016-7606(1987)98<207:COUCTL>2.0.CO;2 (1987).

[58] Perkins, M. E., Nash, W. P., Brown, F. H. & Fleck, R. J. Fallout tuffs of Trapper Creek, Idaho - a record of Miocene explosive volcanism in the Snake River Plain volcanic province. *Geol. Soc. Am. Bull*, 10.1130/0016-7606(1995)107<1484:FTOTCI>2.3.CO;2 (1995).

[59] Pearce NJG, Alloway BV, Westgate JA (2008). Mid-Pleistocene silicic tephra beds in the Auckland region, New Zealand: Their correlation and origins based on the trace element analyses of single glass shards. Quat. Int..

[60] U.S.G.S., Map SS 58-60/2 Cape Hallett, 1:250.000 Antarctica Reconnaisance Series, (1968).

