# Supplementary material for: First marine cryptotephra in Antarctica found in sediments of the western Ross Sea correlates with englacial tephras and climate records
Source: Sci Rep. 2019 Jul 23;9:10628. doi: 10.1038/s41598-019-47188-3 (PMC6650406; doi:10.1038/s41598-019-47188-3)
Supplement: Supplementary file 1 — Supplemental figure 1 [file 41598_2019_47188_MOESM1_ESM.pdf]

First marine cryptotephra in Antarctica found in sediments of the western Ross Sea correlates with englacial tephras and climate records

Alessio Di Roberto[a], Ester Colizza[b], Paola Del Carlo[a], Maurizio Petrelli[c], Furio Finocchiaro[b], Gerhard Kuhn[d]

a - Istituto Nazionale di Geofisica e Vulcanologia, Sezione di Pisa, Via della Faggiola 32, 56126 Pisa, Italy
b - Dipartimento di Matematica e Geoscienze, Università di Trieste, Via E. Weiss 2, 34127 Trieste, Italy
c - Dipartimento di Fisica e Geologia, Università di Perugia, Via A. Pascoli, I-06123, Perugia, Italy
d - Alfred-Wegener-Institut Helmholtz-Zentrum für Polar- und Meeresforschung, Am Alten Hafen 26, D-27568 Bremerhaven, Germany

Supplementary Information

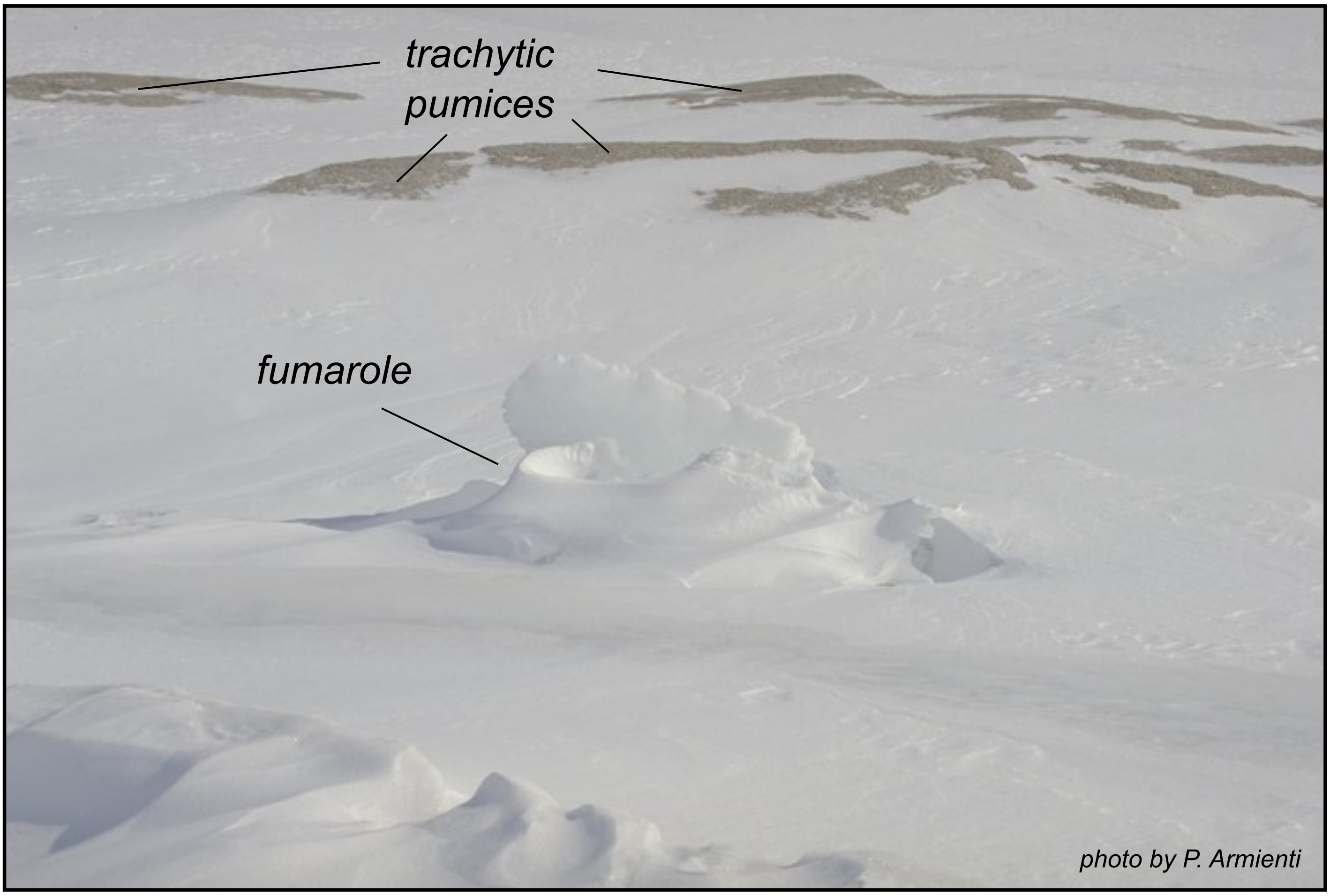

Supplemental Figure 1. Photograph of the Mount Rittmann caldera rim showing active fumarole and lag breccia outcrop where trachytic pumices of sample NN15 have been sampled (for courtesy of Prof. Pietro Armienti)
